# Traumatic Tetanus

**Published:** 1888-07

**Authors:** Bat. Smith

**Affiliations:** Wharton, Texas


### TRAUMATIC TETANUS.

### Anatomical Changes in the Spinal Cord.

*By Bat. Smith, M. D., Wharton, Texas.*

For Daniel's Texas Medical Journal.

OWING to the fact that post-mortems in cases of traumatic tetanus have failed to present constant anatomical changes, we hesitate to pronounce it purely a disease of the nervous system, and many have advanced the hypothesis that traumatic tetanus is dependent on some peculiar blood poison, and not on a lesion of the nerve centers. This view of the pathology of the disease has been, and is yet, held by many occupying exalted positions in the ranks of the profession. Others again, for want of more decided evidences than have as yet been produced by investigators, settle the question at once by falling back upon the very convenient "germ theory;" yet must they admit that their theories are mere speculations, unsupported by facts which can be demonstrated to the satisfaction of the student.

The following cases came under my observation some years ago, and I now submit the result of examinations made at the time:

Case No. 1. Negro boy, age 15 years, strong and stoutly built. Three months before he had stuck a nail in the sole of the left foot, wounding a branch of the internal plantar nerve. The physician attending the case requested me to make the autopsy, which was made at 1 o'clock p. m., the patient having died at 8 o'clock a. m. on the same day. On cutting down to the bottom of the wound, a few drops of pus were found. The wounded nerve was carefully followed up to the interval between the inner malleolus and the heel, at the union of the internal and external plantar nerves with the posterior tibial, and presented nothing abnormal. I was especially particnlar in this part of my examimation, owing to the statement made by the eminent surgeon, John E. Erichsen, viz: "That a marked congestion and inflammation of the nerve connected with, and leading from the wound that has occasioned the disease, always exists." "This morbid state," says he, "I have never found to be wanting. In all fatal cases I have seen, where a careful dissection has been made, the signs of inflammation of a nerve communicating with the wound have been found, and the vascularity, which is intense, may be traced up the membranes, often to a considerable distance."

This constant condition, as reported by the illustrious Englishman, I was unable to find in this case; it was also wanting in the two cases to be mentioned below. The posterior tibial nerve was carefully dissected, and exposed up to the arch of the solius, and no lesions were found; from this point up to the union of the internal and external popliteal nerves with the great sciatic, the condition of the nerves presented nothing abnormal. The trunk of the great sciatic was also found to be in a normal condition, but on arriving at the sacral plexus evidences of a heavy congestion of the first, second, third, and the communicating branch of the fourth anterior divisions of the sacral nerves, and of the lumbo-sacral cord, were plainly visible. In tracing the roots of these nerves to the first lumbar vertebra, the termination of the spinal cord, an intense inflammation, almost amounting to disintegration of the nerve tissue was revealed. An exudation of lymph had taken place over the whole of the dorsal and lumbar region, and the spinal cord from the first lumbar to the eighth dorsal vertebra inclusive, had degenerated into a mass of pus. So far had this disintegration proceeded, that in attempting carefully to lift the spinal cord, the cauda equina, and that portion of the cord above the eighth dorsal, separated from that lying between the eighth dorsal and first lumbar. The disintegration at this point was so great that, upon careful microscopical examination, but little nerve tissue could be recognized. From the eighth to the fifth dorsal, the cord was of a dark red color, giving evidence of an active inflammation, showing in many places extravasation of blood. This inflammation extended to the roots and ganglia of the spinal nerves throughout the cord, which was, however, marked in a much lighter degree between the fifth cervical and the medulla oblongata, amounting here merely to congestion.

I could detect no particular changes in the walls of the blood vessels, except where they had shared in the general disintegration of the surrounding tissues, they were more or less dilated, but not invariably so. From the fifth to the first dorsal the inflamatory condition was not so strongly marked, the cord being of a light rose color, and the dilatation of the arteries not so frequently met with. Again, from the seventh to the fifth cervical the inflammation was more distinct, but not intense as that found between the fifth and eighth dorsal, The cord above the fifth cervical to the medulla oblongata clearly showed a serions congestion, not, however, amounting to inflammation.

The dura mater, arachnoid and pia mater were found in a state of inflammation throughout, except from the fifth cervical to the medulla oblongata. The dura mater was of a light pink color from the medulla down to the fifth cervical, and from there down of a somewhat darker hue.

It will be remembered that this patient had been wounded in the right foot, consequently, as might have been expected, the lesions were more distinct in the right posterior horn, and the right posterior lateral columns of the white matter. Owing to the decussation of the sensory fibres, I found that on the left side the anterior parts of the gray matter were more involved than the right. In the columns of the white matter evidences of hemorrhages were round throughout the whole extent of the cord, except between the medulla oblongata and the fifth cervical vertebra. These hemorrhages were of rare occurrence between the first and fifth dorsal, where the inflammatory process was not so strongly marked. Exudations were found throughout the gray matter, and blood could be pressed out of the cord twelve hours after death; especially was this the case in the gray matter. Brain showed no rigns of inflammatiou or congestion.

Case No. 2. Negro woman, age 60 years; died ten days after receiving the injury which brought on the tetanus, Compound fracture of both bones, just above the ankle. Amputation had been advised, but the attending physician objected. In this case I was also requested to make an autopsy. Unfortunately the wound had been cut into before the examination was made, and, in all probability the nerves at and just above the point of fracture had beed cut away, thus causing the parts to become so much disarranged that a minute and exact examination of the end of the nerve could not be made. The postirior tibial nerve was carefully followed up, as in the first case, with like results. The changes in the sacral plexus and lumbo-sacral cord, as well as those in the spinal cord, were identical with those found in case No. 1. The brain in this case also presented nothing abnormal.

Case No. 3. A mulatto girl, aged 13 years; had been suffering with traumatic tetanus for five days before I saw her. The attack was caused by a splinter entering the plantar region of the foot, wounding a branch of the internal plantar nerve. An incision was made and a splinter, one inch long and about the size of a sulphur match, was removed; this was followed by a few drops of pus. The patient died next day, and the results of the examination in this case were similar to those presented in the two foregoing. I could find no evidences of inflammation of the nerve communicating with the wound, although the examination was made with care. The wound being in the left foot, the anatomical changes were more apparent in the left posterior horn, and left posterior lateral columns of the white matter; and, owing to the decussation of the sensory fibres, on the right side, the anterior parts of the gray matter were more involved than the left.

---

NOTE A—Some years ago, in a conversation with Dr. John H. Bowers, of Columbus, Texas, he related to me a remarkable case of traumatic tetanus. A Mexican had his thumb caught by a rope, the horse pulling back fastened the rope tightly around the thumb. Suddenly the whole skin, flesh and nail were torn away, leaving nothng but the bone. Dr. Bowers, who saw the case immediately afterwards, at once amputated the thumb. Traumatic tetanus set in, in a couple of hours, and the man died a few hours afterwards. The spasms in this case surely were not dependent on the absorption of some poisonous matter, neither can the "germ theory" be here admitted. The spasms were evidently dependent on the morbid excitable condition of the gray matter of the cord, due to the continued irritation of the peripheral nerves.

---

NOTE B.—It was not my inteution to say anything here about the treatment of traumatic tetanus, still, as I have, as yet, not seen the following recommended in any of our text books, I do not think it amiss to refer to it briefly. To control the spasms I have found tobacco, in the form of hypodermic injections of the fluid extract, or of the oil, to answer the purpose, when all other remedies had failed. I remember well the case of an old negro woman, with whom every remedy had proven useless ; the spasms were frequent and terrific ; an injection of four drops of the fluid extract of tobacco, diluted with six drops of water, was administered hypodermically, and a pill containing ¼ of a drop of the oil of tobacco given every 4 hours. The spasms did not return for thirty-six hours ; the injection was then repeated, and kept up for several days at the rate of one injection every twenty-four hours, the patient eventually recovering.

This remedy I have frequently used with horses with the happiest effect in those cases where the attack had been brought on by over-exertion. In traumatic cases with these animals I have not yet used the remedy.

